# Supplementary figures and images for: PopAlu: population-scale detection of Alu polymorphisms
Source: PeerJ. 2015 Sep 22;3:e1269. doi: 10.7717/peerj.1269 (PMC4582951; doi:10.7717/peerj.1269)

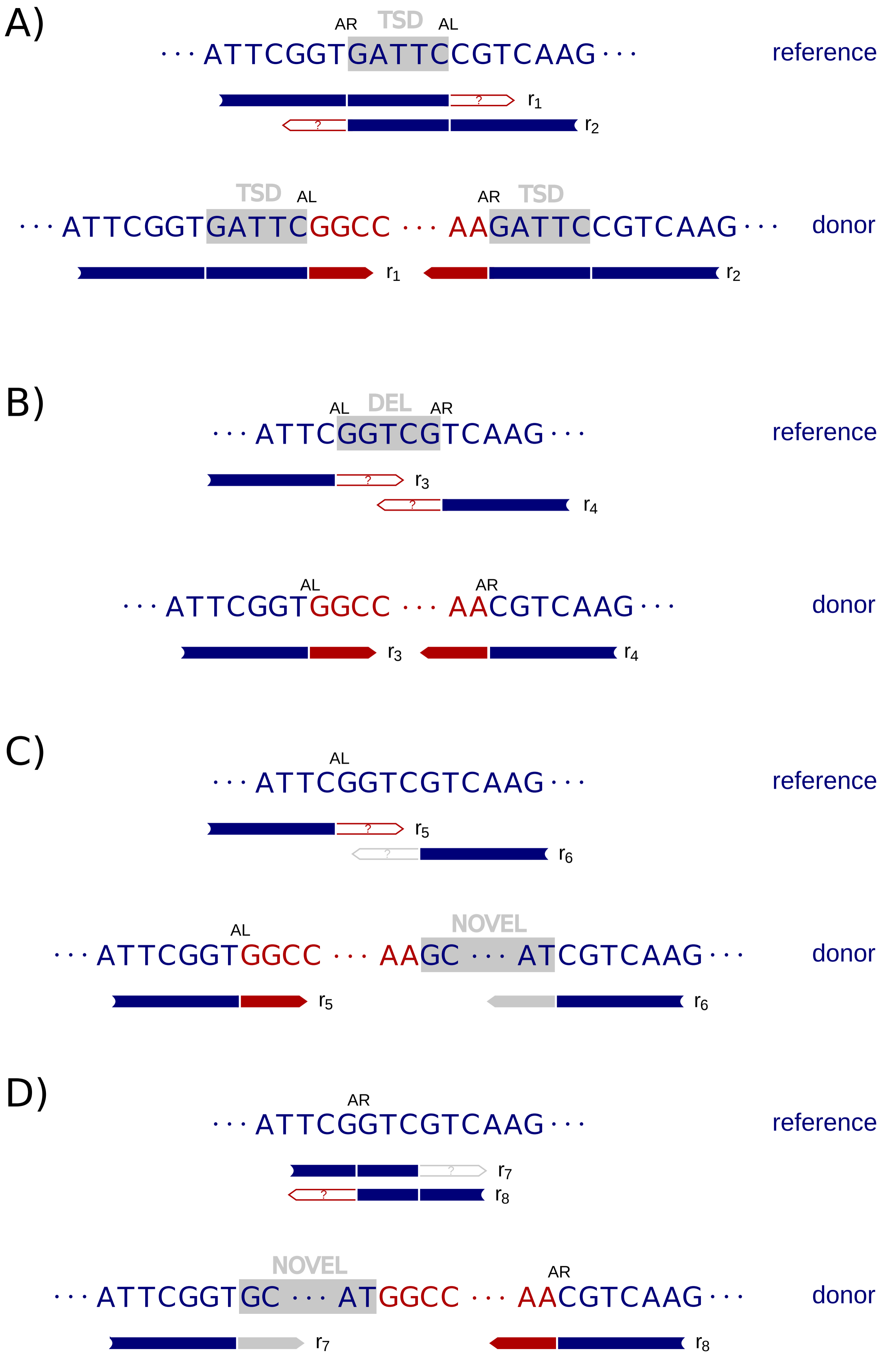

Supplement: Figure S1 — All subfigures show read alignments to the reference (top) and to the donor (bottom). Red letters represent the Alu element. Gray shaded blocks represent either (A) a target-site duplication, (B) a target-site deletion, or (C) and (D) a novel sequence insertion. AL and AR are the breakpoints indicated by split reads mapped to the left and right side of the insertion. We allow the distance between them, |AR − AL|, to range from 0 to 50 bp. In the cases illustrated by subfigures (C) and (D) our algorithm identifies only AL or AR, respectively, not the novel sequence nor the corresponding breakpoint of the novel sequence insertion. [file peerj-03-1269-s001.pdf]
