# Supplementary material for: PopAlu: population-scale detection of Alu polymorphisms
Source: PeerJ. 2015 Sep 22;3:e1269. doi: 10.7717/peerj.1269 (PMC4582951; doi:10.7717/peerj.1269)
Supplement: Figure S2 — The input file chr21_del.fa contains human chromosome 21 with 100 Alu elements deleted, and the input file hap.vcfcontains insertion records for a subset of these regions (see text for details) was chosen between 0 and 199. [file peerj-03-1269-s002.pdf]

```
mason_variator --seed <SEED> --in-reference chr21_del.fa --out-vcf hap.vcf \
--snp-rate 0.0001 --small-indel-rate 0.000001 --max-small-indel-size 6 \
--sv-indel-rate 0 --sv-inversion-rate 0 --sv-translocation-rate 0 \
--sv-duplication-rate 0

mason_simulator --seed <SEED> --read-name-prefix "hap:<SEED>|sim:" \
--num-fragments 2904970 --fragment-mean-size 500 --illumina-read-length 101 \
--illumina-prob-insert 0.0001 --illumina-prob-deletion 0.0001 \
--input-reference chr21_del.fa --input-vcf hap.vcf \
--out reads.1.fastq --out-right reads.2.fastq --out-alignment truth.bam
```
